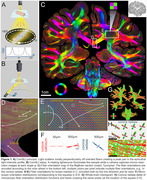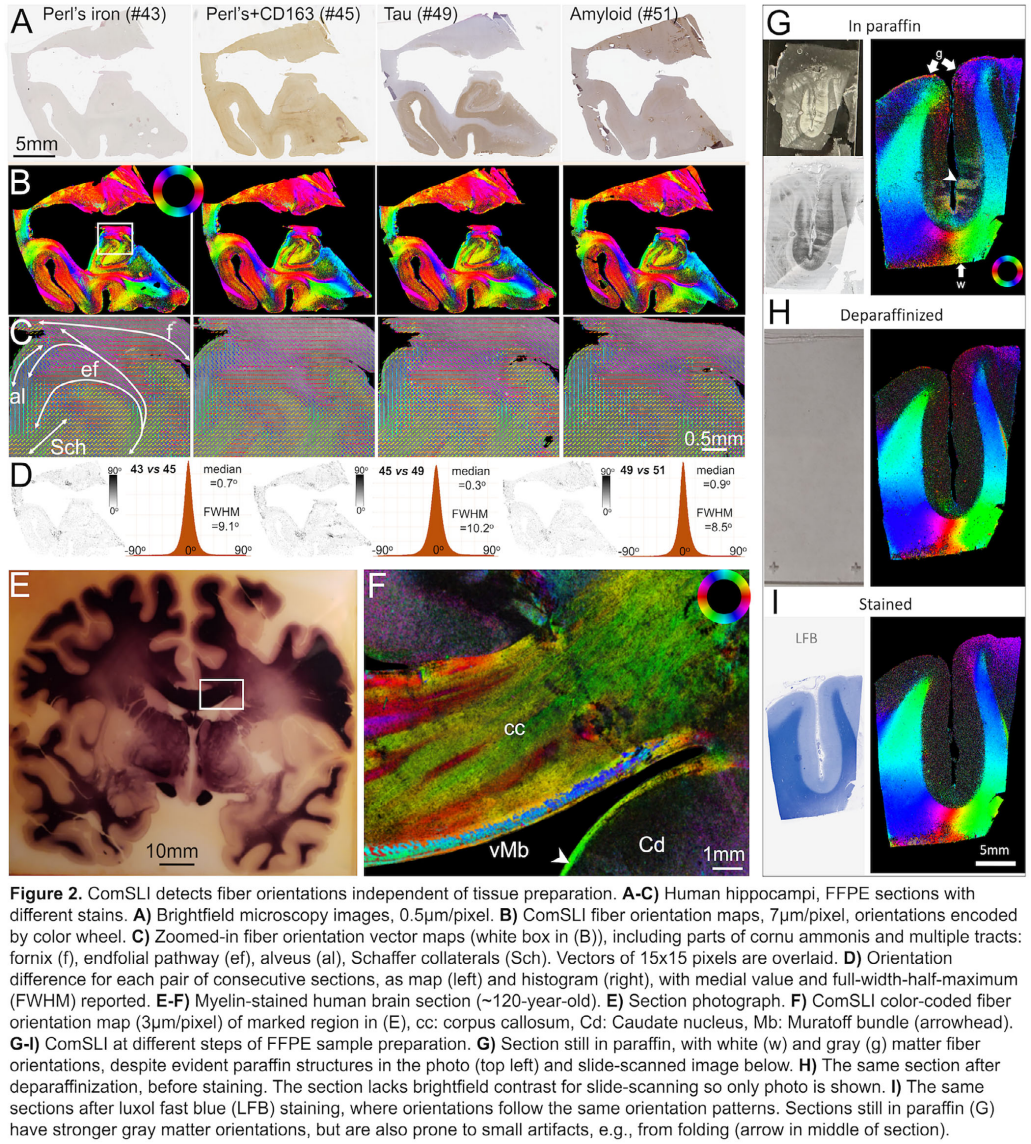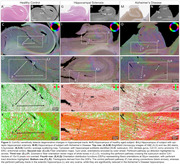# Mapping neuronal trajectories in neurodegeneration independent of sample preparation

**DOI:** 10.1002/alz70855_106440

**Published:** 2025-12-24

**Authors:** Marios Georgiadis, Franca auf der Heiden, Jeffrey Nirschl, Andy Liu, Hossein Moein Taghavi, Katrin Amunts, Markus Axer, Miriam Menzel, Michael Zeineh

**Affiliations:** ^1^ Stanford University, Stanford, CA, USA; ^2^ Forschungszentrum Jülich, Jülich, Germany; ^3^ Institute of Neuroscience and Medicine (INM‐1), Jülich, Germany; ^4^ Forschungszentrum Jülich, Jülich, Germany; ^5^ Delft University of Technology, Delft, Netherlands

## Abstract

**Background:**

The brain's nerve fiber network is disturbed in neurodegeneration, but resolving fiber trajectories over large fields‐of‐view to study connectivity changes remains prohibitive. Current methods study small volumes (electron microscopy), have limited resolution (diffusion MRI), or need birefringence‐preserving sample preparation and cannot resolve crossings (polarization microscopy). Here we show that computational scattered light imaging (ComSLI) resolves neuronal trajectories, including degenerating hippocampal tracts, with micron resolution in any histology section independent of sample preparation.

**Methods:**

We studied standard‐sized 5‐10μm formalin‐fixed paraffin‐embedded (FFPE) sections prepared using various protocols (cf. text/figures) and two whole‐brain sections (Figure 1 – FFPE, from the Jülich BigBrain, 20μm, silver‐stained, and Figure 2E ‐ celloidin‐embedded and myelin‐stained in 1904, from the Institute for Brain Research, Düsseldorf, Germany).

Computational scattered light imaging (ComSLI) was performed in Stanford and Jülich. The setup (Figure 1A,B) includes a micron‐resolution low angle‐of‐acceptance camera‐adapter‐lens system and a rotating LED lightsource. Images were acquired at 5‐15^o^ rotation steps (24‐72 images/sample), with 3‐9μm pixel size. Motorized stages enable tile‐scanning. SLIX software quantified orientations, MATLAB was used for orientation analysis, and MRtrix3 for creating orientation distribution functions and subsequent tractography.

**Result:**

ComSLI produced a micron‐resolution whole‐brain fiber orientation map (Figure 1C). Figures 1D‐E show zoomed‐in fiber orientations in corpus callosum/fornix and corona radiata. Microscopic resolutions enabled generating fiber orientation distributions at multiple scales (Figure 1F), leading to microstructure‐informed whole‐brain tracts (Figure 1G‐H).

ComSLI works for various sample preparation protocols (Figure 2). Consecutive human hippocampal sections with different stains (iron, microglia, tau, and amyloid) show identical orientations (Figure 2B,C), quantified after co‐registration in Figure 2D. Orientations were also derived from a 120‐year‐old human section (Figure 2E‐F), and were consistent at various sample preparation steps (Figure 2G).

ComSLI can study neurodegenerating tracts, such as the hippocampal perforant pathway (Figure 3). A healthy hippocampus includes strong perforant pathway connections through the subiculum and CA1 subfields (Figure 3A‐F), which almost entirely disappear in a sclerotic hippocampus (Figure 3G‐L), and are severely compromised in Alzheimer's disease (Figure 3M‐R).

**Conclusion:**

ComSLI is a cost‐effective method to study intricate fiber networks at micron‐resolution in any histological tissue section, and can reveal subtle changes in neurodegeneration.